# RGB-Style Input Representations for EEG: Evaluating Spatial Concatenation Versus Band-Wise Stacking in Deep Emotion Recognition

**DOI:** 10.3390/brainsci16070716

**Published:** 2026-07-03

**Authors:** Xin Zhang, Ye Li, Fei Pi, Xiu Zhang

**Affiliations:** 1 Tianjin Key Laboratory of Wireless Mobile Communications and Power Transmission, Tianjin Normal University, Tianjin 300387, China; ecemark@tjnu.edu.cn (X.Z.);; 2 College of Artificial Intelligence, Tianjin Normal University, Tianjin 300387, China

**Keywords:** EEG, emotion recognition, multi-feature fusion, convolutional neural network

## Abstract

Background/Objectives: Electroencephalography (EEG) is widely applied in emotion recognition. Integrating diverse frequency and spatial features to improve performance remains a major challenge. Methods: This paper proposes two preprocessing methods to map EEG signals into image-style representations. These methods preserve the spatial topology and enable effective feature extraction using convolutional neural networks. The first method is a spatial concatenation method (SCM). It projects three feature types onto color channels, providing a structural prior that encourages the network to learn the three feature types within local spatial windows. It differs from traditional spectral mixing, which maps frequency bands to color channels. The second method is a band-wise stacking method (BSM). It treats frequency bands as independent depth frames to form a three-dimensional tensor. This structure is designed to facilitate the learning of inter-band relationships while preserving band-specific information. Dedicated convolutional neural network architectures are designed for these tensor structures, aligned with the spatial and spectral organization of the proposed SCM and BSM. Results: Experiments on the DEAP and DREAMER datasets for binary Arousal and Valence classification show that both representations achieve competitive results. The BSM achieves higher accuracy than the SCM on the DREAMER dataset, while both methods perform comparably on the DEAP dataset. Conclusions: The proposed strategies offer efficient convolutional neural network approaches for EEG emotion recognition systems.

## 1. Introduction

With the deep intertwining and fusion of artificial intelligence technology and neuroscience, affective computing has evolved from a theoretical pursuit into a critical technology for next-generation human–computer interaction, precision mental health monitoring, and immersive multimedia experiences [1,2,3]. While exogenous modalities like facial expressions and speech are easily accessible, they are often susceptible to subjective suppression and environmental noise [4,5]. In contrast, electroencephalography, or EEG, which directly captures the electrophysiological dynamics of the central nervous system, offers higher objectivity and temporal resolutions [6,7]. However, unlocking the full potential of EEG for effective emotion recognition remains a formidable challenge, and the demand for high-performance, generalized models has never been greater [8,9].

Despite the rapid advancement of deep learning, or DL, in fields like computer vision, i.e., CV, ref. [10,11], its application to EEG analysis is hindered by a fundamental data–structure mismatch. Unlike images with regular grid structures, EEG signals are high-dimensional, non-stationary time series with complex spatial–spectral couplings [12]. Current research faces a critical bottleneck: traditional methods relying on handcrafted features often fail to capture global dependencies, while end-to-end deep models may require additional structural guidance to converge on raw chaotic signals. Furthermore, relying on a single feature dimension is insufficient to describe the intricate neural mechanisms of human emotion, leading to reduced reliability in complex scenarios [13]. Therefore, how to effectively translate heterogeneous EEG features into a unified, structured representation that is compatible with standard deep neural networks is a pivotal problem that needs immediate resolution.

To address these challenges, this paper proposes a framework based on “RGB-style” multimodal feature construction. We argue that a key factor for performance improvement lies not just in deeper networks but in a more expressive and structured input representation.

We introduce two feature construction strategies: the spatial concatenation method and the band-wise stacking method, abbreviated as SCM and BSM, respectively. Instead of treating features in isolation, we comprehensively extract three feature descriptors—the power spectral density, differential entropy, and spectral strength, i.e., PSD, DE, and SS—and reorganize them into high-dimensional tensors. PSD and DE are classic spectral features that are widely validated in EEG emotion recognition, characterizing the energy distribution and signal complexity, respectively. SS is a more recent spectral measure that captures the cumulative spectral magnitude within each frequency band, and it has shown effectiveness in reflecting emotion-related oscillatory changes. By fusing these three feature types within structured topological priors, this approach transforms the abstract EEG emotion recognition problem into a standard image classification task. The SCM creates a multi-feature image to facilitate local cross-modal fusion, while the BSM treats frequency bands as independent depth frames to facilitate the learning of inter-band dependencies.

While prior work [12] has primarily mapped distinct frequency bands to RGB channels, this paper utilizes the structured fusion of heterogeneous feature types. The primary contributions of this paper are centered on investigating structural priors for multi-feature EEG fusion. Specifically, the novelty of this work is summarized in the following aspects.

(1) Feature-to-channel mapping strategy: Whereas previous RGB-style approaches map different frequency bands to RGB channels, this work proposes a mapping strategy that projects heterogeneous feature types onto the color channels. This structural prior is designed to encourage the convolutional neural network to learn three feature types within local spatial windows, which is distinct from the spectral mixing in traditional methods.
(2) Band-wise stacking method: The band-wise stacking method is a tensor-based strategy that treats frequency bands as independent depth frames. This structure provides a structural pathway for the network to learn band-specific patterns by organizing each frequency band into a separate channel, in contrast to standard representations where band information is combined within the same tensor.
(3) Dedicated convolutional neural network architectures: Two specialized convolutional neural network architectures are designed to be structurally compatible with the spatial and spectral tensor structures generated by the SCM and BSM. These architectures are compact and interpretable, optimized for the specific structural priors imposed by the fusion methods.

The remainder of the paper is organized as follows. Section 2 introduces related work about feature extraction, spatial topology modeling, and deep learning architectures. Section 3 presents the proposed SCM and BSM with a focus on structural design and applicability analysis. Section 4 describes the dedicated CNN architectures. Section 5 gives the experimental configuration. Section 6 presents and analyzes the experimental results and comparative performance of the SCM and BSM. Section 7 concludes the paper and proposes future research directions.

## 2. Related Work

This section critically reviews the technical evolution in EEG emotion recognition, focusing on three key dimensions: feature extraction, spatial topology modeling, and deep learning architectures. We position the proposed framework against the closest related work and clarify where existing methods fall short.

### 2.1. EEG Feature Extraction and Multi-Feature Fusion

Feature extraction is the bedrock of EEG analysis. Early research primarily focused on handcrafted features [14]. Ref. [15] established the utility of PSD in analyzing energy distributions, and it remains one of the most widely adopted frequency-domain features in EEG analysis. Subsequently, ref. [16] proposed DE and demonstrated that it performs competitively with—and, in some settings, achieves higher accuracy than—PSD in classifying varying emotional states due to its sensitivity to signal complexity. Both PSD and DE are well-established, effective EEG descriptors that capture EEG aspects like the energy distribution and information-theoretic complexity, respectively. Ref. [13] further investigated critical frequency bands, identifying that high-frequency bands, specifically β and γ, contain more emotion-related information. PSD and DE have since become standard features in the EEG emotion recognition literature, owing to their well-understood physiological interpretations and computational simplicity.

More recently, SS has emerged as an alternative frequency-domain descriptor besides PSD and DE features. While PSD averages the power within each band and DE captures log-variance, SS sums the raw FFT magnitudes across the frequency components of the band-pass-filtered signal. This provides a distinct measure of the cumulative spectral intensity within each band, making it sensitive to overall oscillatory strength changes induced by emotional arousal. Its integration alongside PSD and DE enables the more complete characterization of EEG spectral conttent compared to any single feature alone.

Moreover, relying on a single feature type is often insufficient to capture the full spectrum of brain dynamics. Recent trends have shifted towards multi-feature fusion [17]. For instance, ref. [18] utilized ensemble learning to combine multi-scale frequency bands, improving stability. Ref. [19] proposed the regularized deep fusion of kernel machines to integrate multimodal signals. Despite these advances, a critical limitation of existing fusion approaches is that they typically concatenate feature vectors into a flat representation, discarding the spatial topology of the electrode layout and the structural relationships among features. Ref. [20] similarly noted the pitfalls of high-dimensional concatenation without structural priors. To address this gap, our work introduces a structured fusion strategy that integrates energy features, namely PSD and SS, and complexity features, namely DE, into a unified tensor, preserving their distinct characteristics within a spatially organized grid.

### 2.2. Input Representation: From Signals to RGB-Style EEG Images

Traditional methods treat EEG signals as one-dimensional time series, or 1D vectors, often discarding critical spatial information. To leverage the feature extraction capabilities of CV, researchers have pioneered the concept of the “RGB-style” EEG image.

The seminal work in [12] proposed projecting three-dimensional electrode coordinates, i.e., 3D spatial positions, onto a two-dimensional, i.e., 2D, plane using azimuthal equidistant projection, or AEP for short. In this paradigm, the spatial distribution of electrodes corresponds to pixel positions, and the spectral power of distinct frequency bands, specifically θ,α,β, is mapped to the standard red, green, and blue color channels, or RGB for short. This transformation effectively converts the emotion recognition problem into an image classification task, allowing the model to capture local spatial patterns. While this paradigm has been successfully applied, most existing RGB-style EEG images map different frequency bands to the three color channels. In contrast, the present work explores a different direction: mapping different feature types extracted from the same set of frequency bands to the RGB channels. Through this, we investigate whether the convolutional neural network, or CNN, can learn three feature types within a local spatial window, rather than purely spectral mixing. This mapping strategy provides a structural prior that allows the CNN to jointly process multiple feature descriptors within the same spatial context.

Parallel to image-based methods, graph neural networks, or GNNs, have also emerged as an effective tool for modeling spatial topology. Ref. [21] proposed the dynamical graph convolutional neural network, i.e., DGCNN, to dynamically learn the intrinsic relationships between channels. More recently, ref. [22] introduced a hierarchical dynamic GCN with interpretability, and ref. [23] surveyed the growing application of GNNs in this field. For cross-subject generalization, Wang et al. proposed a meta-learning wavelet graph convolutional network [24]. Beyond single-modal EEG, Yan et al. combined frequency-domain graph convolution and time-domain convolution with cross-attention for EEG-fNIRS fusion [14].

While GNNs are effective, image-based representations offer a practical advantage: they enable the seamless transfer of mature, pretrained CNN backbones such as ResNet and EfficientNet to EEG tasks. The SCM proposed in this paper extends the RGB-style EEG image concept. Whereas ref. [12] maps frequency bands to color channels, the SCM maps heterogeneous feature types to the RGB channels. This mapping allows the CNN to learn interactions between different feature modalities within the same spatial receptive field.

### 2.3. Deep Modeling Architectures

With the evolution of input representations, backend models have diversified. Convolutional neural networks, or CNNs, remain dominant; for example, ref. [25] designed EEGNet, a compact CNN specifically tailored to BCI, while refs. [26,27] explored 3D-CNNs to simultaneously capture spatial and temporal–spectral dependencies.

Recently, transformer architectures have gained traction due to their ability to model long-range dependencies. Refs. [28,29] successfully integrated attention mechanisms with convolutional networks. Ref. [30] proposed an adaptive multiview fusion transformer, and ref. [31] developed ERTNet to enhance model interpretability. Furthermore, ref. [32] explored advanced transformations for deep learning, and ref. [33] extended recognition to continuous regression tasks. For feature refinement, Zhang et al. introduced a triple attention network to fuse temporal, spatial, and cross-modal features [34]. To capture spatial dependencies, Zhu et al. constructed distinct spatial maps for different frequency bands using self-organized graph pseudo-3D convolution [35]. Fang et al. encoded band–space–time correlations into 3D tensors and utilized a multi-scale CNN with bidirectional GRUs [36].

Building on these foundations, this paper proposes the BSM as a tensor-based deep modeling strategy. Whereas standard 2D-CNNs process a single fused input, the BSM treats frequency bands as independent depth frames, allowing the model to learn cross-band correlations. Furthermore, while GNNs and transformers have shown promise in modeling the spatial topology and long-range dependencies, they often rely on complex attention mechanisms or graph constructions. Our proposed dedicated CNN architectures are compact, interpretable, and specifically designed to exploit the structural priors embedded in our input representations, rather than relying on generic deep learning backbones.

## 3. Methodology

### 3.1. Overall Process Overview

**Figure 1:**
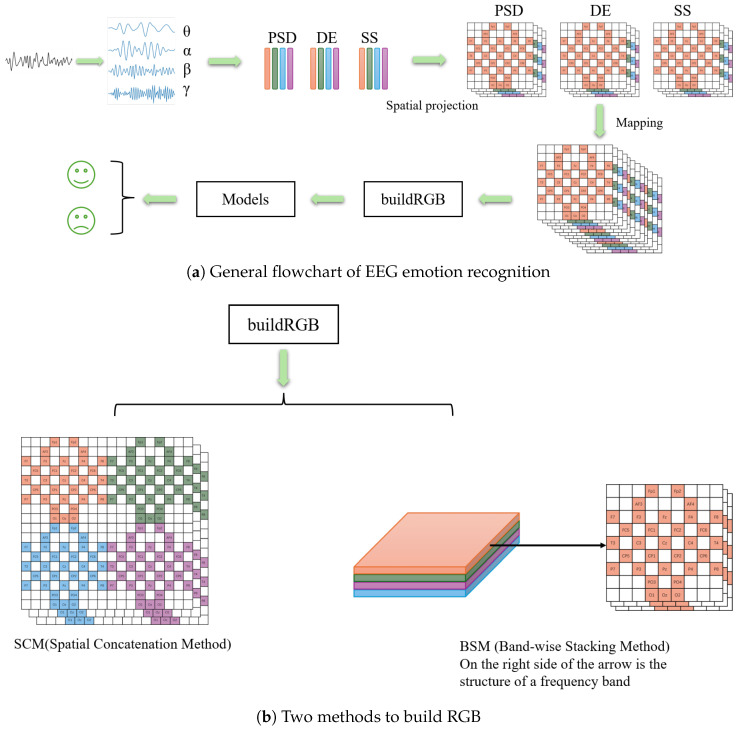
The overall research pipeline of the proposed EEG emotion recognition framework.

This work aims to enhance the fusion and representation capabilities of multi-band, multi-feature information for EEG emotion recognition models by designing rational feature construction strategies. As illustrated in Figure 1, the overall research pipeline is structured into five consecutive stages: EEG signal preprocessing, feature extraction, feature construction based on the proposed SCM and BSM strategies, deep model training and inference, and, finally, comprehensive performance evaluation.

In the first stage, the raw EEG signals undergo preprocessing operations such as filtering, artifact removal, and segmentation to eliminate noise and ensure data comparability across different subjects. Subsequently, in the second stage, three types of commonly used frequency-domain and spatial features are calculated for each segmented sample: DE, PSD, and SS. These features characterize emotion-related EEG patterns from perspectives such as energy distribution, information complexity, and spatial topology. Normalization is applied after feature extraction, as described in Section 3.3.

The third stage is the core aspect of this work: the feature construction stage. In this stage, the three extracted feature types are reorganized into high-dimensional tensor inputs compatible with deep learning networks. The two proposed methods in this paper are as follows:

(1) The spatial concatenation method concatenates PSD, DE, and SS along the channel dimension to construct an input structure similar to an RGB image and further arranges them along the spatial or frequency band axis to achieve local three-feature fusion.
(2) The band-wise stacking method constructs a three-channel feature map for each frequency band individually and then stacks multiple bands along an added dimension, forming a multi-frame input to facilitate learning dependencies between frequency bands along the band dimension.

Through this multi-source feature and structured tensor mapping, the EEG spectral and spatial information is converted into a unified format. This format could be directly used in 2D or 3D convolutional networks. While previous approaches performed numerical concatenation of features, this design preserves multi-dimensional structural information, allowing subsequent models to extract local and cross-band patterns.

In the fourth stage, the features generated by the SCM and BSM are input into various backend models for training and inference, including 2D-CNNs, 3D-CNNs, and hybrid architectures incorporating attention mechanisms. By using uniform training parameters and evaluation strategies, a fair comparison of different representation methods under equivalent conditions is ensured. The final stage involves the performance evaluation and ablation experiments, systematically analyzing the two methods across multiple dimensions, such as accuracy, the F1-score, generalizability, and computational costs.

Overall, the methodological framework establishes a tight connection between the feature layer and the model layer: the frontend SCM/BSM is responsible for structured feature representation, and the backend network is responsible for feature learning and classification. This complete “feature construction–model adaptation–performance verification” closed loop allows us to systematically investigate the impact of EEG feature organization on emotion recognition performance at the input level.

### 3.2. EEG Signal Preprocessing

For both the DEAP and DREAMER datasets, we directly used the official preprocessed versions provided by the dataset authors. According to the original papers [37,38], these versions already include downsampling to 128 Hz; a common average reference, i.e., CAR; re-referencing; ocular artifact removal via ICA or regression; and band-pass filtering (4–45 Hz). No additional artifact rejection or baseline correction was performed by us.

On top of the official preprocessing, we applied the following steps before feature extraction.

- Butterworth filtering: A fourth-order zero-phase Butterworth filter was applied to further isolate the four frequency bands of interest, as listed in Table 1. The filtering range is set to 4–45 HZ to retain information in the main frequency bands [39]. The zero-phase filtering prevents phase shift.
- Segmentation: Each trial was divided into non-overlapping 0.5 s windows. No overlap was used between adjacent windows to avoid temporal leakage.

The filtered EEG signal is further decomposed into multiple frequency bands for subsequent feature extraction and indexing operations. This paper adopts the frequency band division scheme shown in Table 1.

The δ band (0.5–4 Hz) is excluded from the experiments for two reasons. First, the δ band predominantly reflects slow-wave activity associated with deep sleep and unconscious states, and it has been shown to carry limited discriminative information for emotion recognition compared to the higher-frequency bands (θ, α, β, γ) [13]. Second, the official preprocessed versions of both DEAP and DREAMER are band-pass-filtered to 4–45 Hz [37,38], which already attenuates the δ band content. All subsequent processing therefore operates on the four bands within the 4–45 Hz range.

### 3.3. Feature Extraction

To extract multi-dimensional emotion-related information from the preprocessed EEG signals, this paper utilizes three features: PSD, DE, and SS. These three features describe brain activity from the perspectives of energy distribution, signal complexity, and spatial topology, respectively. These three feature types collectively support the subsequent feature construction methods, namely the SCM and BSM, in fully integrating cross-band and cross-channel discriminant information. This section details the calculation method and output format for each feature type.

After feature extraction, z-score normalization is applied to the extracted features to eliminate inter-subject differences and channel amplitude biases. Specifically, for each subject and each of the three feature types, namely PSD, DE, and SS, the mean and standard deviation are computed per channel across all windows, and the normalization is then applied to the corresponding feature values:

(1) $${\tilde{f}}_{i}=\frac{f_{i}-\mu_{i}}{\sigma_{i}}$$

where $f_{i}$ denotes the extracted feature value (PSD, DE, or SS) of channel *i*, and $\mu_{i}$ and $\sigma_{i}$ are the per-channel mean and standard deviation. For the subject-dependent protocol, the normalization statistics are computed exclusively on the training folds, and the resulting $\mu_{i}$ and $\sigma_{i}$ are then applied to normalize both the training and test folds, ensuring that no information from the test set is involved in the normalization. For LOSO experiments, the normalization statistics are similarly computed on the training subjects only and applied to the held-out test subject. This normalization is performed on the extracted features, not on the raw EEG signals, thereby preserving the amplitude and energy characteristics that PSD, DE, and SS are intended to capture during the feature extraction stage.

#### 3.3.1. Power Spectral Density

The PSD feature describes the energy distribution of the EEG signal at different frequencies and is one of the most commonly used frequency-domain features in emotion recognition research. In this paper, the Welch method [15] is utilized to estimate the spectrum of the segmented signal for each channel. Specifically, the calculation process begins by dividing the signal of length *T* into several overlapping sub-windows, followed by the application of windowing and the fast Fourier transform, i.e., FFT, on each segment. Finally, the energy within each frequency band interval is computed and averaged to obtain the stable spectral density.

Its mathematical expression is as follows:

(2) $${\mathrm{PSD}}_{c,b}=\frac{1}{N_{w}}\sum_{n=1}^{N_{w}}\left(\sum_{f\in F_{b}}{\left|P_{c,n}\left(f\right)\right|}^{2}\right)$$

where *c* denotes the channel number; *b* denotes the frequency band, θ,α,β, or γ; $N_{w}$ is the number of sub-windows; and $F_{b}$ is the set of frequencies corresponding to band *b*.

#### 3.3.2. Differential Entropy

The DE feature reflects the complexity of the signal from an information theory perspective and is considered to be closely related to changes in neural activity associated with emotion regulation. Since EEG can be approximated as a Gaussian process over a short period, if the signal follows $X∼N(0,\sigma^{2})$, its differential entropy can be written as [16]

(3) $$DE_{c,b}\left(X\right)=\frac{1}{2}\log\left(2\pi e\sigma_{c,b}^{2}\right)$$

where $\sigma_{c,b}^{2}$ denotes the variance of the band-pass-filtered signal for channel *c* and frequency band *b*. In this paper, the variance is computed from the corresponding signal segment within each channel *c* and each frequency band *b* to obtain the DE feature. Compared to PSD, the DE feature is more sensitive to amplitude and statistical fluctuations, providing additional information beyond pure energy.

#### 3.3.3. Spectral Strength

To further capture the spectral intensity characteristics of the EEG signal within each frequency band, this paper introduces the SS feature. While PSD estimates the average power and DE captures the signal complexity, SS directly quantifies the cumulative spectral magnitude, providing an additional perspective on the overall oscillatory strength within a given frequency range.

The calculation is performed on the band-pass-filtered signal for each frequency band. Specifically, for a single-channel EEG segment $x_{c}\left(t\right)$ that has been band-pass-filtered to a target band, the FFT is computed and the magnitudes of the positive frequency components are summed:

(4) $${\mathrm{SS}}_{c,b}=\sum_{k=0}^{N/2-1}\left|F{\left\{x_{c,b}\left(t\right)\right\}}_{k}\right|$$

where $x_{c,b}\left(t\right)$ denotes the channel-*c* signal after band-pass filtering to band *b*, *N* is the number of time-domain samples, and $F{\left\{x_{c,b}\left(t\right)\right\}}_{k}$ is the *k*-th complex FFT coefficient. Unlike PSD and DE, which characterize specific statistical properties (mean power and log-variance, respectively), SS captures the aggregate spectral activity through the sum of the raw FFT magnitudes. We note that SS is related to the signal amplitude and spectral energy; rather than claiming independence among PSD, DE, and SS, we view SS as an additional, practically effective EEG descriptor whose combination with the other two features, through the RGB-style stacking framework, contributes to improved classification performance.

Concerning the scale dependence of the raw FFT magnitude, we note that per-subject z-score normalization is applied after feature extraction, as detailed in Section 3.3, which mitigates inter-subject amplitude differences by standardizing each feature to a common scale within each subject. The relative magnitude across frequency bands within a single subject is preserved after this normalization.

Similarly, to maintain consistency with PSD and DE, the SS feature for each frequency band is mapped onto a 2D electrode plane via the same topology-preserving mapping described above, allowing it to participate in the spatial/band structural construction of the SCM and BSM.

#### 3.3.4. Electrode Topology Mapping

To construct the 2D grid-like representations required by the SCM and BSM, the multi-channel EEG feature vectors are projected onto a fixed 9×9 spatial grid. This mapping is performed via a predefined template that assigns each electrode to a specific (row, column) position based on its approximate scalp location, following the international 10–20 system layout. Positions without corresponding electrodes are filled with zeros.

**Figure 2:**
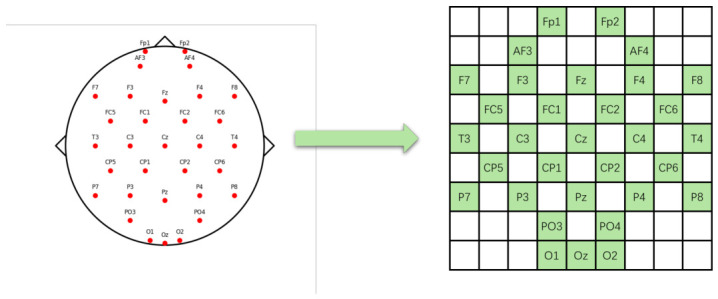
Electrode-to-grid mapping template for the DEAP dataset with 32 channels. Each electrode is assigned to a fixed position on the 9×9 grid. Empty cells are zero-padded.

For the DEAP dataset, the 32 electrode indices are placed at coordinates approximating their standard 10–10 positions on the 9×9 grid. For the DREAMER dataset, the 14-channel layout is similarly mapped to the same 9×9 grid with zero padding at unused positions. Compared to some prior work that employs azimuthal equidistant projection, i.e., AEP, for coordinate-based interpolation, the template-based approach used here directly assigns each electrode’s scalar feature value to a fixed grid cell, which is computationally efficient and preserves the discrete spatial relationships among neighboring electrodes without introducing interpolation artifacts. The resulting output for each feature type and frequency band is a 9×9 matrix, which serves as a single-channel spatial map. The electrode-to-grid coordinate mapping for the DEAP 32-channel layout is illustrated in Figure 2. The DREAMER 14-channel layout follows the same mapping procedure.

### 3.4. Feature Construction Methods

After completing the preprocessing of the EEG signals and the extraction of multiple features, effectively organizing these heterogeneous features into an input representation that can be fully utilized by deep learning models becomes a crucial factor affecting the emotion recognition performance. Addressing this issue, this paper proposes two structured feature construction methods: the SCM and the BSM. The two methods differ in their focus regarding feature fusion, frequency band modeling strategies, and model adaptability, catering to different application requirements.

#### 3.4.1. Spatial Concatenation Method

(1) Design Motivation

The core idea of the SCM is to closely fuse multiple feature types within the spatial dimension, creating an input structure that is analogous to an RGB image. This allows CNNs to directly learn multiple feature correlations within the local receptive field. This method emphasizes “local properties between features”. It is suitable for standard 2D-CNN frameworks and enhances the discriminative ability without increasing the network complexity.

(2) Construction Process

The construction process begins with selecting the target frequency bands, denoted as $idx_{band}$, from the extracted PSD, DE, and SS features. For each selected frequency band, these three feature types are concatenated along the channel dimension, i.e., axis=1, to generate a unified three-channel feature block:

(5) $$F_{b}=\mathrm{Concat}\left({\mathrm{PSD}}_{b},{\mathrm{DE}}_{b},{\mathrm{SS}}_{b}\right)\in R^{H\times W\times 3}$$

When the selection involves multiple frequency bands, the corresponding feature blocks are spatially arranged to form a composite map. Specifically, in a dual-band configuration, the blocks are concatenated along the height direction, whereas, in a four-band configuration, they are organized into a 2×2 grid array. The final output is a single 2D feature map, which serves as the direct input for the standard 2D-CNN.

**Figure 3:**
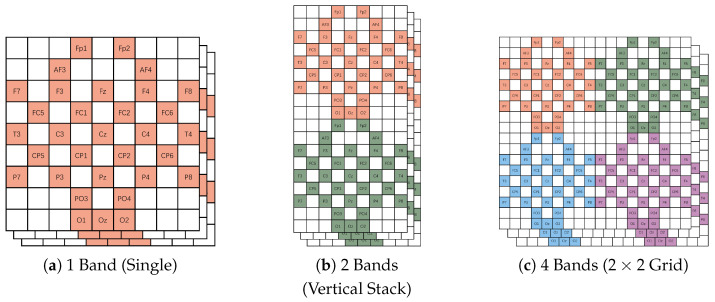
Illustration of SCM spatial arrangement strategies for different numbers of frequency bands. (**a**) Single band directly used as input; (**b**) two bands concatenated vertically; (**c**) four bands arranged in a 2×2 grid to form a larger feature map.

As illustrated in Figure 3, this structure can be regarded as a “colored EEG image”, where each pixel simultaneously contains three types of information: PSD, DE, and SS. The tensor shape is given in Table 2, where *N* denotes the batch size. The SCM thus requires a four-dimensional input.

(3) Advantage Analysis

The SCM strategy presents several practical characteristics. It fuses multiple feature types within local spatial regions, providing multiple-feature information within the receptive field. Additionally, it is compatible with standard 2D-CNN architectures without requiring structural modifications, making it suitable for scenarios where computational efficiency is important.

#### 3.4.2. Band-Wise Stacking Method

(1) Design Motivation

The BSM focuses on the structural relationships and cross-band dependencies between frequency bands. This method treats different frequency bands as independent “frames”, preserving their distinctiveness, and stacks them along a newly added dimension, allowing the model to learn interaction patterns between bands.

(2) Construction Process

The construction process of the BSM is executed in a structured sequence. Initially, for each frequency band *b*, the corresponding features from PSD, DE, and SS are extracted separately. These three feature types are then concatenated along the channel dimension to form a unified single-band feature block:

(6) $$F_{b}=\mathrm{Concat}\left({\mathrm{PSD}}_{b},{\mathrm{DE}}_{b},{\mathrm{SS}}_{b}\right)\in R^{H\times W\times 3}$$

Subsequently, all generated band feature blocks are stacked along a newly introduced frequency band dimension, corresponding to axis=1:

(7) $$I_{\mathrm{BSM}}=\mathrm{Stack}\left(F_{b_{1}},F_{b_{2}},\ldots ,F_{b_{k}}\right)\in R^{k\times H\times W\times 3}$$

where *k* denotes the number of bands. The resulting structure constitutes the final five-dimensional tensor input to the model, typically processed with shape N×k×H×W×3, where *N* denotes the batch size.

The output shapes for different frequency band settings are shown in Table 3. It can be seen that the BSM needs a 5D input.

**Figure 4:**
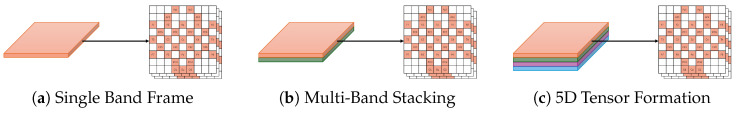
Illustration of the BSM structure. On the right side of the arrow is the structure of a single frequency band. (**a**) Feature extraction for a single band; (**b**) stacking multiple bands as independent frames; (**c**) the resulting high-dimensional tensor input.

Figure 4 illustrates the BSM structure. In this figure, each frequency band is a frame of a three-channel feature map, stacked along the band axis to form a 3D tensor structure.

(3) Structural Characteristics

The BSM strategy has several structural characteristics. Primarily, it organizes each frequency band as a separate frame, which allows the network to process each band through dedicated branches before fusing the extracted features. Furthermore, the method is adaptable to various frequency band combinations. Finally, on the datasets evaluated, this design shows competitive performance in the LOSO evaluation compared to single-stream methods.

## 4. Dedicated CNN Architectures

To evaluate the SCM and BSM in the EEG emotion recognition task, this paper designs two adapted CNN architectures: a custom convolutional neural network called CustomCNN and a parallel four-branch convolutional neural network called ParallelCNN. Both are designed to be consistent with the characteristics of the SCM and BSM in terms of the input structure, convolution method, and feature fusion mechanism, so that the models are compatible with the tensor structures formed by the two construction methods.

### 4.1. CustomCNN Model Architecture

(1) Adaptability Design

The SCM construction method spatially concatenates the three feature types into a single three-channel EEG image with the dimension

(8) $$I_{\mathrm{SCM}}\in R^{H^{\prime }\times W^{\prime }\times D}$$

where D=3 for the three feature types. This structure is naturally compatible with traditional 2D-CNNs; thus, this paper uses CustomCNN to process the SCM data.

(2) Network Structure

**Figure 5:**
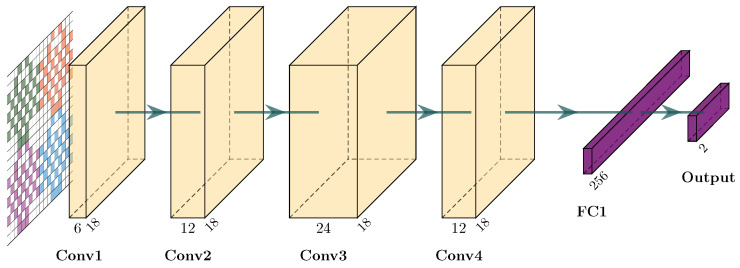
Structure of the CustomCNN model.

The structure of CustomCNN is shown in Figure 5. CustomCNN consists of four consecutive convolutional blocks, achieving gradual abstraction from the local spatial structure to higher-order semantic features, followed by fully connected layers for classification. Specifically, the network employs 3×3 convolutional kernels with the channel dimensions progressively expanding (3→16→32→64→128). Each convolutional operation is immediately followed by a ReLU activation function. Notably, pooling layers are intentionally omitted throughout the network to avoid the loss of fine-grained spatial information and to maintain the integrity of the generated EEG topological maps. Following the feature extraction stage, the resulting high-dimensional tensors are flattened into vectors and fed into a classification head consisting of fully connected layers (Flatten→256→num_classes). This design balances parameter efficiency with representational power for processing the compact spatial structures generated by the SCM strategy. The forward propagation flow of the CustomCNN model is as follows:

(9) $$X\overset{\mathrm{Conv}}{\to }F_{\mathrm{Conv}}\overset{\mathrm{Flatten}}{\to }f_{\mathrm{Flat}}\overset{\mathrm{FC}}{\to }y$$

The convolutional layers are responsible for learning the fusion patterns across feature channels, and the final fully connected layer performs classification prediction.

### 4.2. ParallelCNN Model Architecture

(1) Adaptability Design

The BSM construction method preserves frequency band independence by treating each band as a separate frame of a three-channel feature map, stacked along a new dimension:

(10) $$I_{\mathrm{BSM}}\in R^{N_{band}\times H\times W\times D}$$

where $N_{band}$ denotes the number of frequency bands. Specifically, when $N_{band}=4$, the BSM forms four complete feature frames. To fully utilize these structural characteristics, we designed ParallelCNN, a network composed of four parallel branches that independently model the features of the θ, α, β, and γ bands.

(2) Network Structure and Forward Propagation

**Figure 6:**
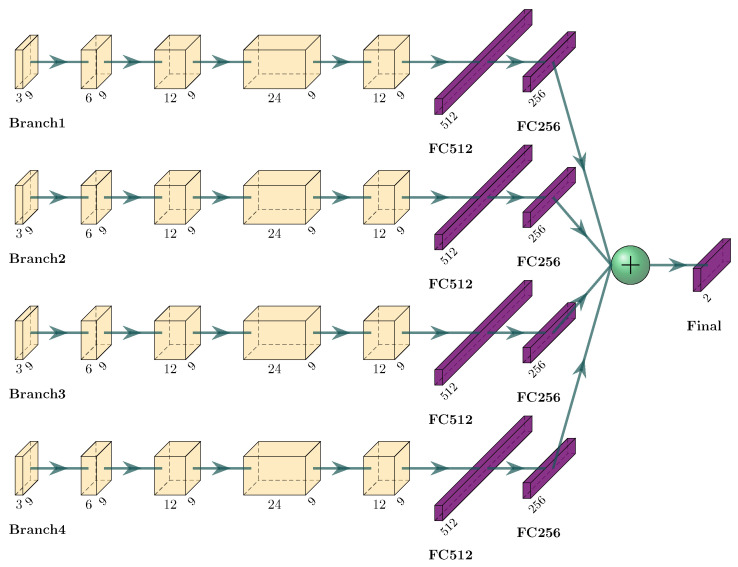
Structure of the ParallelCNN model.

The structure of ParallelCNN is shown in Figure 6. The architecture consists of four structurally identical convolutional branches. Each branch processes its corresponding input $X_{b}\in R^{H\times W\times 3}$ (where b∈{θ,α,β,γ}) through a series of operations: multi-layer convolutions using 3×3 kernels, with the channel dimensions progressively expanding (3→16→32→64→128), followed by ReLU activation and batch normalization. The resulting feature maps are then flattened and passed through two fully connected layers (512→256) to extract high-level representations.

These independent representations are subsequently fused at the feature level via concatenation:

(11) $$f_{\mathrm{fused}}=\mathrm{Concat}\left(\mathrm{Branch}\left(X_{\theta }\right),\ldots ,\mathrm{Branch}\left(X_{\gamma }\right)\right)$$

Finally, the fused feature vector $f_{\mathrm{fused}}$ is fed into a linear classifier to generate the final prediction y.

This multi-branch design processes each frequency band through a dedicated branch with independent weights, so that features from different frequency ranges are initially processed separately. The late fusion strategy then combines these representations to learn cross-band correlations.

### 4.3. Model Complexity and Applicability Comparison

The two models are designed to be structurally compatible with the SCM/BSM construction methods, so that the different feature organization approaches can be evaluated, with neither approach inherently superior to the other as shown in Table 4.

We note that CustomCNN and ParallelCNN are architecturally coupled to their respective representation strategies: CustomCNN is a single-branch architecture tailored to the three-channel input format, combining PSD, DE, and SS, produced by the SCM, while ParallelCNN is a four-branch architecture where each branch independently processes one frequency band of the BSM tensor. Direct cross-pairing, such as pairing the SCM with ParallelCNN or the BSM with CustomCNN, is architecturally non-trivial: feeding the 3-channel SCM tensor into ParallelCNN would collapse its four branches into a single branch, losing the structural purpose of the parallel design; conversely, using the BSM with CustomCNN would require replicating CustomCNN into four independent copies—one per band—and fusing their outputs, which essentially yields a variant of ParallelCNN. Therefore, the evaluation of the architecture-agnostic baselines—EEGNet, DeepConvNet, and ShallowConvNet—under both the SCM and BSM provides a controlled comparison between representation strategies, while the dedicated architectures, CustomCNN and ParallelCNN, are evaluated only within their structurally compatible representations. This inherent coupling between representation and architecture is acknowledged as a limitation of the current study; full cross-factorial disentanglement is deferred to a future investigation.

## 5. Experimental Configuration

### 5.1. Experimental Framework

To systematically evaluate the SCM and BSM feature construction methods proposed in the task of EEG emotion recognition, this section presents a complete experimental pipeline. Comparative and ablation experiments are conducted on the basis of unified data preprocessing, feature extraction, and model architectures.

#### 5.1.1. Overview of Experimental Procedure

The experimental procedure of this paper is systematically structured into five core stages. Initially, the raw EEG signals undergo data preprocessing, involving a series of standard cleaning operations such as artifact removal, band-pass filtering, re-referencing, and windowed segmentation. During this phase, different frequency bands are isolated based on predefined indices to lay the foundation for subsequent processing. Subsequently, the process moves to multi-feature extraction, where three features are computed to capture diverse aspects of brain activity. Specifically, PSD is extracted to describe the energy distribution, DE characterizes the signal complexity, and SS represents the cumulative spectral magnitude within each frequency band. All three features are uniformly mapped onto the same 2D spatial topology to ensure consistency.

Based on these extracted features, the procedure advances to the feature construction stage. Here, the PSD, DE, and SS features are fused into structured input representations using two distinct strategies. The SCM concatenates features in the spatial dimension to construct a three-channel feature map, analogous to an RGB image. In contrast, the BSM stacks features along a new dimension corresponding to frequency bands, constructing a 3D feature tensor optimized for cross-band modeling.

With the structured inputs prepared, the experiment proceeds to model training, where inputs from the SCM and BSM are trained separately using 2D-CNN and parallel CNN backbones, respectively. Uniform hyperparameter settings are strictly maintained across all experiments to ensure comparability. Finally, the study concludes with a performance evaluation on the test set. The classification performance is assessed using the following metrics. Accuracy (12) measures the overall proportion of correctly classified samples. The F1-score (13) provides a balanced assessment by computing the harmonic mean of the precision and recall, which is particularly informative for class-imbalanced scenarios. Balanced accuracy (BA) (14) is the arithmetic mean of the per-class recall rates, mitigating the influence of class imbalance. The macro F1-score (MF1) (15) computes the F1-score independently for each class and then averages them, giving equal weight to each class regardless of its size. The weighted F1-score (WF1) (16) averages the per-class F1-scores weighted by the number of true instances per class. The area under the ROC curve (AUC) (17) evaluates the model’s ability to discriminate between classes across all classification thresholds.

(12) $$\mathrm{Accuracy}=\frac{TP+TN}{TP+TN+FP+FN}$$

(13) $$F1\text{-}\mathrm{score}=2\times \frac{\mathrm{Precision}\times \mathrm{Recall}}{\mathrm{Precision}+\mathrm{Recall}}$$

where $\mathrm{precision}=\frac{TP}{TP+FP}$ and $\mathrm{recall}=\frac{TP}{TP+FN}$.

(14) $$\mathrm{BA}=\frac{1}{2}\left(\frac{TP}{TP+FN}+\frac{TN}{TN+FP}\right)$$

(15) $$\mathrm{MF}1=\frac{1}{C}\sum_{i=1}^{C}{F1}_{i}$$

(16) $$\mathrm{WF}1=\sum_{i=1}^{C}w_{i}\cdot {F1}_{i},\;w_{i}=\frac{N_{i}}{N_{\mathrm{total}}}$$

where *C* is the number of classes and $N_{i}$ is the number of samples in class *i*.

(17) $$\mathrm{AUC}=\int_{0}^{1}\mathrm{TPR}\left({\mathrm{FPR}}^{-1}\left(t\right)\right)\;dt$$

where TPR, or the true positive rate, and FPR, or the false positive rate, are computed at varying decision thresholds. Additionally, confusion matrices are visualized to analyze the per-class prediction patterns.

#### 5.1.2. Experimental Objectives and Validation Strategy

The experimental design is centered around three core objectives. First, to evaluate the proposed SCM and BSM, we compare their recognition performance under identical model configurations, systematically analyzing how their structural differences relate to emotion recognition performance. Second, we investigate the influence of frequency information by setting up various single-band, dual-band, and multi-band combinations to analyze the sensitivity of the task to band selection. Finally, to assess the contribution of multi-feature fusion, we conduct ablation experiments by selectively removing PSD, DE, or SS features, evaluating the individual contribution of each feature type and thereby examining the combined utility of the proposed SCM and BSM strategies.

### 5.2. Experimental Settings

To evaluate the proposed SCM and BSM feature construction methods in the EEG emotion recognition task, this section introduces the dataset, data partitioning method, hyperparameter settings, and experimental environment used in the experiments.

#### 5.2.1. Dataset Introduction

This study is a secondary analysis of publicly available, de-identified datasets. Ethical approval and informed consent for data collection were obtained in the original studies.

This paper uses two internationally widely adopted public affective computing datasets: DEAP [37] and DREAMER [38].

The DEAP dataset consists of EEG recordings from 32 participants, each watching 40 emotional video stimuli. The data were collected using 32 EEG channels at a sampling rate of 128Hz. Each trial lasts 60s, including a 3s pretrial baseline. Similarly, the DREAMER dataset includes EEG data from 23 participants, recorded using a 14-channel portable Emotiv EPOC device at a sampling rate of 128Hz while they watched 18 film clips. Participants in DREAMER rated their emotions on a scale of 1 to 5. After watching the stimuli, participants in both datasets provided self-assessments across dimensions including Arousal, Valence, and Dominance [40].

This paper focuses on the most commonly used Arousal/Valence binary classification tasks. Binary labels are constructed by binarizing the scores according to dataset-specific thresholds—score ≥5 for DEAP and score >3 for DREAMER—following mainstream research practice. Table 5 reports the window-level class distribution for each dataset and emotion dimension. DEAP Arousal, with C0:C1 approximately 41:59, and Valence, at approximately 43:57, exhibit moderate imbalance, while DREAMER shows a stronger skew, particularly for Arousal, with C0:C1 approximately 28:72. These distributional characteristics should be considered when interpreting the accuracy, as the metric may be inflated by a model’s tendency to predict the majority class. We therefore report the balanced accuracy, macro F1-score, and AUC alongside the accuracy in all experiments to provide a more complete picture of the model performance.

Classification performance is assessed using the accuracy, balanced accuracy, macro F1-score, weighted F1-score, and area under the ROC curve, abbreviated as BA, MF1, WF1, and AUC, respectively, as defined in Section 5.1.1.

#### 5.2.2. Data Preprocessing and Segmentation Strategy

As described in Section 3.2, we adopted the official preprocessed versions of DEAP and DREAMER. Our own preprocessing consisted of three steps. First, fourth-order Butterworth band-pass filtering was applied to isolate the target frequency bands: θ at 4–8 Hz, α at 8–13 Hz, β at 13–30 Hz, and γ at 30–45 Hz, with the full 4–45 Hz band-pass already applied by the dataset authors. The δ band is not used in subsequent processing, as explained in Section 3.3. Second, the signals were segmented into non-overlapping 0.5 s windows, with no overlap between adjacent windows. Third, per-subject z-score normalization was applied to the extracted features, PSD, DE, and SS; for each subject and each feature type, the mean and standard deviation are computed per channel across all windows. For the subject-dependent protocol, normalization statistics are computed exclusively on the training folds and applied to both training and test folds; for LOSO, statistics are computed on the training subjects only and applied to the held-out test subject.

The generated samples are subsequently used for feature extraction and the construction of the SCM and BSM.

#### 5.2.3. Training and Test Set Split

Two validation strategies are adopted to evaluate model performance from different perspectives. One is trial-wise 5-fold cross-validation, abbreviated as CV; the other is leave-one-subject-out cross-validation, or LOSO.

Trial-wise 5-fold cross-validation: To evaluate the within-subject recognition performance, a trial-wise stratified 5-fold cross-validation protocol is adopted as shown in Algorithm 1. For each subject, all 40 trials from DEAP or 18 trials from DREAMER are partitioned into five folds at the trial level via stratified splitting based on trial-level class labels, ensuring that all 0.5 s windows belonging to the same trial are kept entirely within the same fold. In each fold, one subset is held out as the test set, while the remaining four subsets serve as the training set; the process is repeated five times with each fold used exactly once for testing. This trial-wise splitting prevents windows from the same trial from appearing in both the training and test sets, thereby avoiding inflated performance estimates due to temporal leakage. The reported metrics are the averages over the five folds. Identical fold division is applied to all models to ensure a fair comparison.    
**Algorithm 1:** Trial-wise stratified 5-fold cross-validation under the subject-dependent protocol  **Input**   : EEG data of all subjects; number of folds K=5; random seed  **Output**: Per-subject cross-validated classification metrics 
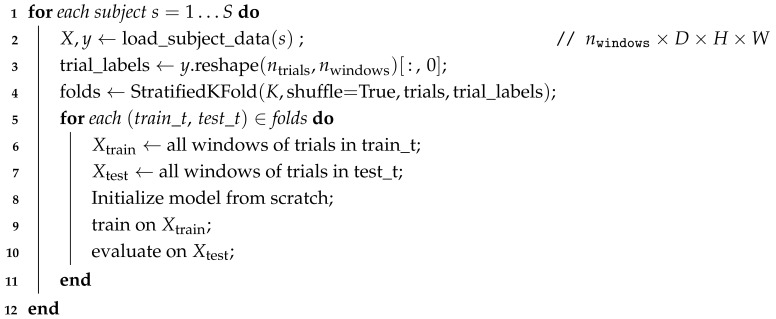


Leave-one-subject-out (LOSO) cross-validation: To evaluate the subject-independent generalization capability, a LOSO protocol is implemented on both the DEAP and DREAMER datasets. In the LOSO setup, the data from one subject are entirely held out as the test set, while the model is trained on the data from all remaining subjects. This procedure is repeated for all subjects, and the final performance metrics are obtained by averaging across all folds. LOSO strictly ensures that the test subject is completely unseen during training, thereby providing a realistic assessment of model generalization to new users.

The training hyperparameters were predetermined prior to all experiments and are listed in Table 6. No hyperparameter search, including grid search, random search, or Bayesian optimization, was conducted. The same set of hyperparameters was applied uniformly across all datasets, i.e., DEAP and DREAMER; all emotion dimensions, i.e., Arousal and Valence; and both cross-validation protocols.

All random sources were fixed with seed 2025, including Python’s random, NumPy 1.24.3, PyTorch CPU 2.1.0, and CUDA 11.8 generators and the random_state of StratifiedKFold. cuDNN deterministic mode was enabled, and benchmark mode was disabled to ensure bitwise reproducibility. All models were trained from scratch with randomly initialized weights for each cross-validation fold.

No validation set was held out from the training data. Early stopping was not employed; all models were trained for a fixed number of epochs, as specified in Table 6. During trial-wise 5-fold cross-validation, a stratified split was applied at the trial level.

Importantly, the test fold was used exclusively for evaluation and was never involved in model selection or hyperparameter tuning. The same fixed hyperparameter configuration was used across all subjects, folds, datasets, and emotion dimensions. For the subject-dependent experiments, the per-subject results are reported as the average over the five folds.

## 6. Results and Discussion

In this section, we systematically evaluate the proposed SCM and BSM feature construction strategies on the DEAP and DREAMER datasets. The experiments were organized into four parts: first, an ablation study on the multi-feature fusion effectiveness; second, a comprehensive comparison between the SCM and BSM across multiple architectures with confusion matrix analysis; third, a model complexity and efficiency analysis; and, fourth, cross-subject generalization via LOSO validation. All results are reported as the mean ± standard deviation across subjects.

### 6.1. Ablation Study: Multi-Feature Fusion Effectiveness

To assess the contribution of each feature type and the benefit of multi-feature fusion, we conducted ablation experiments using both CustomCNN with the SCM and ParallelCNN with the BSM on the DEAP Arousal task under the trial-wise five-fold CV protocol. Table 7 summarizes the results across five input configurations: the full RGB fusion combining PSD, DE, and SS; three single-feature configurations using DE only, PSD only, or SS only; and a raw PSD baseline without multi-feature concatenation, labeled no RGB.

For CustomCNN in its single-branch configuration, when a single feature such as PSD only is used, the input is a single-channel spatial map without any feature concatenation along the channel dimension—i.e., SCM-style multi-feature fusion is not applied. Consequently, the PSD-only and no-RGB entries are identical for CustomCNN. For ParallelCNN in its multi-branch configuration, the single-feature cases such as PSD only still process each of the four frequency bands through an independent branch, preserving the BSM’s structural design; the no-RGB configuration, by contrast, concatenates the four-band PSD values into a single four-channel tensor and processes it through a single-branch architecture. Thus, the ablation design implicitly captures not only the contributions of each feature type but also, through the CustomCNN PSD-only and no-RGB conditions, the baseline performance without the proposed multi-feature representation.

Several observations emerge from Table 7. First, the full RGB fusion, i.e., PSD+DE+SS, achieves the best mean values across all metrics for both architectures under the DEAP Arousal task. This suggests that stacking multiple well-established EEG features into a multi-channel input can yield better classification performance than using any single feature alone. We do not claim that PSD, DE, and SS are statistically independent or strictly complementary; rather, the results indicate that each feature captures partially distinct signal characteristics that, when combined within the RGB-style framework, collectively benefit the CNN’s discriminative capability. Second, among the single-feature configurations, PSD alone performs the best across most metrics, followed by SS and DE, consistent with the established role of PSD as a robust frequency-domain descriptor in EEG analysis. Third, the no-RGB configuration yields identical results to PSD only for CustomCNN since the input dimensions coincide for single-branch models, with slightly higher results for ParallelCNN, suggesting that the four-band multi-frame structure itself may offer a modest benefit even without feature fusion. Fourth, both SCM+CustomCNN and BSM+ParallelCNN perform competitively under the full RGB configuration—CustomCNN achieves comparable results with higher parameter efficiency, while ParallelCNN attains slightly higher balanced accuracy and F1-scores. These results indicate that both representation strategies are viable for this task. However, this ablation study is limited to a single dataset and classification task, namely DEAP Arousal under the subject-dependent protocol, and the contributions of individual features may vary across other datasets, emotion dimensions, and evaluation protocols. The generalizability of the multi-feature fusion benefit therefore requires further verification across a wider range of conditions.

### 6.2. Comprehensive Model Comparison

This section presents the core comparison between the SCM with CustomCNN and BSM with ParallelCNN, alongside three standard EEG baselines—EEGNet [25], DeepConvNet [41], and ShallowConvNet [41]—evaluated under both the SCM and BSM input representations. Results are reported for both the DEAP and DREAMER datasets under two protocols: trial-wise five-fold CV for the subject-dependent setting and LOSO for the subject-independent setting.

Several findings emerge from the subject-dependent results in Table 8. First, both the proposed SCM+CustomCNN and BSM+ParallelCNN combinations achieve competitive performance across all metrics on both datasets. Under the SCM, CustomCNN achieves the best scores on most but not all metrics: EEGNet attains a slightly higher AUC on DEAP Valence at 0.6990 versus 0.6977, and ShallowConvNet achieves a higher AUC on DREAMER Valence at 0.8364 versus 0.8344. Under the BSM, ParallelCNN achieves the best scores on most DEAP Valence and DREAMER settings; however, on DEAP Arousal, EEGNet edges ahead in Acc at 0.7417 versus 0.7411 and DeepConvNet leads in BA and MF1 at 0.6450 and 0.6369 versus 0.6345 and 0.6281. Second, among the baseline architectures, EEGNet delivers competitive results on DEAP across most metrics. ShallowConvNet shows competitive performance on DREAMER, achieving an Arousal BA of 0.8669 under the SCM and an MF1 of 0.8336 under the BSM, but it trails on DEAP. DeepConvNet under the BSM achieves the best BA at 0.6450 and MF1 at 0.6369 on DEAP Arousal. This pattern suggests that the optimal architecture can vary with the dataset characteristics and that no single model dominates all metrics. Third, both the SCM and BSM representations yield broadly comparable performance. On DEAP, BSM+ParallelCNN achieves moderately higher balanced accuracy and F1-scores than SCM+CustomCNN on both Arousal and Valence; on DREAMER, BSM+ParallelCNN leads on most metrics, although SCM+CustomCNN achieves a slightly higher MF1 on Valence at 0.8197 versus 0.7962. Without statistical significance testing, these numerical differences should be interpreted as descriptive trends rather than confirmed advantages. Overall, the results indicate that both proposed strategies are viable for subject-dependent EEG emotion recognition, each with different structural characteristics.

**Figure 7:**
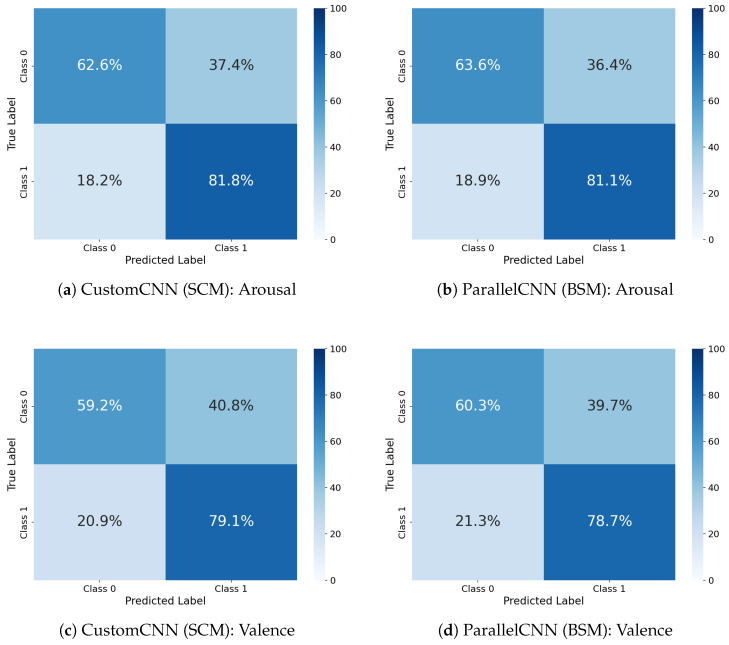
Confusion matrices on the DEAP dataset under the subject-dependent protocol. Left: SCM+CustomCNN; Right: BSM+ParallelCNN. Top: Arousal; Bottom: Valence. Matrices show predictions aggregated across all subjects from one selected fold of the 5-fold CV.

**Figure 8:**
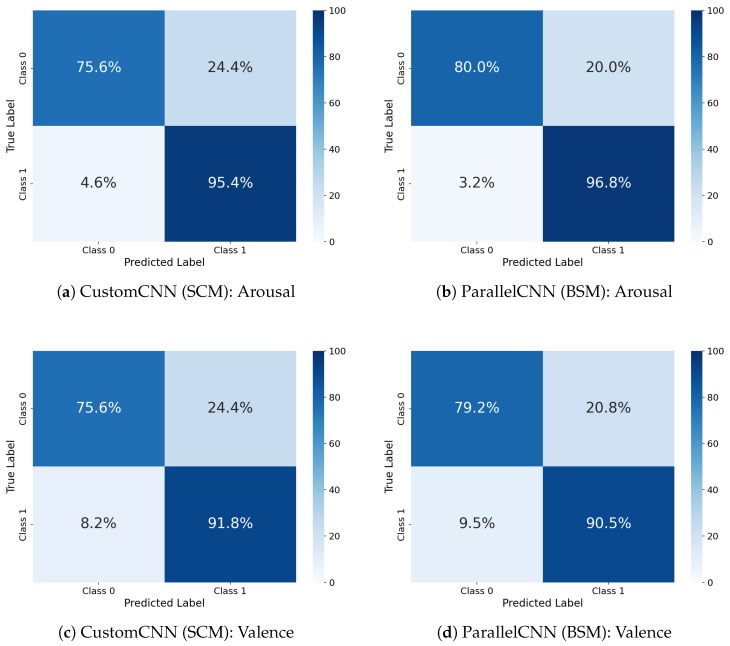
Confusion matrices on the DREAMER dataset under the subject-dependent protocol. Left: SCM+CustomCNN; Right: BSM+ParallelCNN. Top: Arousal; Bottom: Valence. Matrices show predictions aggregated across all subjects from one selected fold of the 5-fold CV.

To complement the quantitative metrics, confusion matrices for the proposed SCM+CustomCNN and BSM+ParallelCNN pipelines are presented for the DEAP dataset in Figure 7 and for the DREAMER dataset in Figure 8, both under the subject-dependent protocol. Since trial-wise five-fold cross-validation produces five sets of predictions per subject, the confusion matrices shown are aggregated across all subjects for one selected fold.

On the DEAP dataset, as shown in Figure 7, both SCM+CustomCNN and BSM+ParallelCNN exhibit diagonal dominance in the selected fold, suggesting that the models are capable of distinguishing between classes above the chance level. On the DREAMER dataset, as shown in Figure 8, both pipelines also achieve visible diagonal patterns, particularly for the Arousal task. The confusion matrices, based on one selected fold out of the five CV folds, indicate that both the SCM and BSM representations, when paired with their respective dedicated architectures, can learn class-discriminative patterns. These visual patterns are broadly consistent with the quantitative results in Table 8, although the per-fold results exhibit some variation across the five splits.

### 6.3. Model Complexity and Efficiency

Table 9 reports the parameter count, FLOPs, and per-sample inference time for all evaluated models, measured on an NVIDIA RTX 4070 Ti SUPER. Both single-branch SCM-compatible variants and four-branch BSM-compatible variants are included.

Several observations are noteworthy. Since the memory consumption is uniformly minimal across all compared methods and is approximately 1 MB, we omit it from the table as it provides no meaningful discriminative information. First, EEGNet is the most lightweight model, with only 1.6 K parameters in the single-branch configuration, approximately 1/683 of CustomCNN’s 1.09 M. In the four-branch variant, EEGNet has only 2.9 K parameters, about 1/878 of ParallelCNN’s 2.54 M. Second, despite their larger parameter counts, CustomCNN and ParallelCNN achieve competitive inference speeds, namely 0.26 ms and 1.54 ms, respectively, due to their purely Conv2d+Linear architecture, which avoids depthwise-separable convolutions or other computationally intensive operations. Third, ShallowConvNet is the fastest model overall at 0.19 ms in single-branch and 0.71 ms in four-branch configurations, with minimal GPU memory of 9.07 MB for single-branch and 10.00 MB for four-branch, making it an attractive option for latency-critical and memory-constrained deployments. Fourth, the four-branch models scale approximately 4× in parameters and FLOPs compared to their single-branch counterparts, as each branch independently processes one frequency band without parameter sharing. DeepConvNet has the highest computational cost among the baselines, at 0.0021 GFLOPs in single-branch and 0.0069 GFLOPs in four-branch configurations. Finally, in terms of the model storage size, CustomCNN and ParallelCNN are notably larger, at 4.17 MB and 9.71 MB, respectively, than the lightweight alternatives, such as EEGNet at 0.01 MB, which may be relevant for on-device deployment with storage constraints. Overall, on the datasets evaluated, CustomCNN and ParallelCNN achieve competitive accuracy with inference latency that is sufficient for offline batch processing on GPU hardware, albeit at the cost of higher parameter counts and storage requirements than ultra-lightweight alternatives.

### 6.4. Cross-Subject Generalization: LOSO Validation

To evaluate generalization to unseen subjects, LOSO experiments were conducted on both the DEAP and DREAMER datasets. Table 10 presents the aggregated LOSO results for all models under both SCM and BSM representations.

The LOSO results in Table 10 reveal several patterns. First, there is a substantial performance drop from subject-dependent to subject-independent evaluation across all models and datasets, underscoring the inherent difficulty of cross-subject EEG emotion recognition and indicating that the highly subject-dependent results are partly attributable to subject-specific patterns rather than fully generalizable emotion representations.

Second, both the SCM and BSM representations yield broadly comparable results under LOSO, although the highest-scoring method varies by dataset, task, and metric. On DEAP Arousal, SCM+CustomCNN achieves the best BA and MF1 at 0.5163 and 0.5003, respectively, while BSM+ParallelCNN leads in Acc at 0.5887. On DEAP Valence, the best results are distributed across models: BSM+DeepConvNet attains the highest BA and AUC at 0.5372 and 0.5525, while SCM+CustomCNN achieves the highest MF1 at 0.5263 and BSM+ParallelCNN leads in Acc at 0.5740. On DREAMER, BSM+ParallelCNN achieves the top mean values across all four metrics for both Arousal and Valence. However, many metrics are close to the chance level with BA near 0.5, particularly on DEAP, and the large standard deviations suggest that these numerical differences should be interpreted cautiously as descriptive trends only. No formal statistical test was applied to compare model pairs at the subject level, so differences across models should not be interpreted as statistically confirmed advantages. No single architecture or input representation consistently outperforms all others across all conditions.

Third, the inter-subject variability is considerably larger under LOSO than under the subject-dependent evaluation, and the DREAMER dataset exhibits wider variance than DEAP, likely reflecting the smaller subject sample and the different electrode montage. This variability highlights that domain adaptation or personalization strategies remain an important area for future work before these models can be reliably deployed on unseen users.

Fourth, the discrepancy between the accuracy and balanced metrics, particularly evident under LOSO, is partly attributable to class imbalance in the datasets. As reported in Table 5, DEAP shows moderate imbalance, with Arousal C0:C1 approximately 41:59 and Valence approximately 43:57, while DREAMER exhibits a stronger skew, with Arousal C0:C1 approximately 28:72 and Valence approximately 39:61. A model that tends to predict the majority class can achieve inflated accuracy under such distributions, which explains the gap between the accuracy, balanced accuracy, and macro-F1 observed in the LOSO results. This observation further reinforces the importance of evaluating EEG emotion recognition models with multiple metrics, as the accuracy alone can be misleading when the class distributions are not perfectly uniform.

## 7. Conclusions

In this paper, we have presented a systematic study on constructing input representations for EEG-based emotion recognition. To address the high dimensionality and complex spatial–spectral structure of EEG signals, we propose the SCM and BSM feature construction strategies. They reorganize PSD, DE, and SS into structured tensors suitable for CNN processing. The proposed methods were evaluated through experiments on the DEAP and DREAMER datasets for binary Arousal and Valence classification, under both subject-dependent and subject-independent protocols. The following findings are drawn.

(1) Multi-feature fusion is beneficial. The ablation study reported in Table 7 shows that integrating PSD, DE, and SS into an RGB-style representation achieves higher accuracy than any single feature modality on the DEAP Arousal task. The fusion improves the accuracy by approximately 7–12 percentage points over the best single feature, PSD. Rather than claiming strict complementarity or independence among features, we interpret this as evidence that stacking multiple well-established EEG descriptors into a multi-channel input provides richer discriminative information for CNN-based classification than any single feature alone. However, this finding is based on a single dataset and task, namely DEAP Arousal under the subject-dependent protocol, and the contribution of each feature type may vary under different conditions and evaluation settings.
(2) Both the SCM and BSM are viable representation strategies. Across both datasets and evaluation protocols, the proposed SCM+CustomCNN and BSM+ParallelCNN pipelines achieve competitive performance. On DEAP, the two approaches yield comparable results: subject-dependent Valence Acc of 0.7058 versus 0.7086 and Arousal Acc of 0.7405 versus 0.7411. On DREAMER, BSM+ParallelCNN yields higher numerical scores for most metrics, while SCM+CustomCNN achieves a higher MF1 on Valence, 0.8197 versus 0.7962, with a more compact architecture. The confusion matrix analysis based on one selected CV fold, shown in Figure 7 and Figure 8, demonstrates that both representations can produce diagonally dominant predictions. These findings suggest that the choice between the SCM and BSM may be informed by application-specific constraints such as model complexity, inference latency, or whether inter-band modeling is desired. However, without statistical significance testing, numerical comparisons should be interpreted as indicative rather than definitive.
(3) CustomCNN and ParallelCNN offer a competitive accuracy–efficiency trade-off. While EEGNet is orders of magnitude smaller at 1.6 K versus 1.09 M parameters for CustomCNN, the proposed architectures achieve the highest numerical scores in most subject-dependent settings, with inference latencies of 0.26 ms for CustomCNN and 1.54 ms for ParallelCNN per sample on a commodity GPU, specifically an NVIDIA RTX 4070 Ti SUPER. Under LOSO, their performance is more mixed, with no single model consistently leading across all metrics. The GPU memory footprints of the proposed models remain low at 12.53 MB for CustomCNN and 17.91 MB for ParallelCNN, although their model storage sizes of 4.17 MB and 9.71 MB are notably larger than EEGNet’s 0.01 MB. This suggests that reasonably compact, purely convolutional architectures can support EEG emotion recognition without relying on pretrained heavyweight backbones, although lighter alternatives may be preferable under strict resource constraints.
(4) Cross-subject generalization remains a major challenge. The LOSO experiments reveal a substantial performance gap between subject-dependent and subject-independent evaluation, i.e., approximately 14–25 percentage points in accuracy depending on the dataset and emotion dimension, and large inter-subject variability, with standard deviations ranging from roughly 4% to 13%. These findings highlight the need for domain adaptation, personalization, or data augmentation strategies in future EEG emotion recognition research.

Future work will explore three directions: first, conducting experiments on additional datasets to further evaluate the proposed methods; second, incorporating graph neural networks to replace the fixed 2D grid mapping with learnable, non-Euclidean electrode connectivity; and, third, investigating domain adaptation and self-supervised pretraining techniques to narrow the subject-dependent-to-LOSO performance gap.

## Figures and Tables

**Table 1:** Frequency band division scheme.

Band Name	Frequency Range (Hz)
θ (Theta)	4–8
α (Alpha)	8–13
β (Beta)	13–30
γ (Gamma)	30–45

**Table 2:** The relationship between the number of $idx_{band}$s and the SCM output shape.

${\mathrm{idx}}_{\mathrm{band}}$	SCM Output Shape
1	N×3×H×W
2	N×3×2H×W
4	N×3×2H×2W

**Table 3:** Output shapes of BSM with different numbers of frequency bands.

${\mathrm{idx}}_{\mathrm{band}}$	BSM Output Shape
1	N×1×3×H×W
2	N×2×3×H×W
4	N×4×3×H×W

**Table 4:** Comparison of model complexity and applicability between CustomCNN and ParallelCNN.

Feature Method	Model Type	Input Dimension	Specialization
SCM	CustomCNN	$R^{H^{\prime }\times W^{\prime }\times 3}$	Spatial Local Features, Cross-Feature Fusion
BSM	ParallelCNN	$R^{N_{band}\times H\times W\times 3}$	Band-Level Patterns, Inter-Band Relationships

**Table 5:** Window-level class distribution for DEAP and DREAMER datasets.

Dataset	Emotion	Ratio (C0:C1)
DEAP	Arousal	41.1%:58.9%
	Valence	43.4%:56.6%
DREAMER	Arousal	27.5%:72.5%
	Valence	38.9%:61.1%

**Table 6:** Hyperparameter settings and reproducibility details.

Parameter/Item	Value/Configuration
Optimizer	Adam
Learning rate	$1\times 10^{-3}$
Batch size	128
Number of epochs	300
Loss function	Cross-entropy
CV folds	5
Split strategy	Trial-wise 5-fold/LOSO
Weight init (Conv/Linear)	Kaiming Uniform
Weight init (BatchNorm)	Weight = 1, bias = 0
Dropout (CustomCNN)	0 (no dropout)
Dropout (ParallelCNN)	0.5
Dropout (baseline CNNs)	0.5
BatchNorm (CustomCNN)	None
BatchNorm (ParallelCNN)	BatchNorm2d
Random seed (global)	2025
Random seeds (Python/NumPy/PyTorch)	2025
cuDNN deterministic	True
cuDNN benchmark	False
Early stopping	Not used
Validation set	Not used
Hyperparameter search	None
GPU	NVIDIA RTX 4070 Ti SUPER

**Table 7:** Ablation study of feature configurations on the DEAP Arousal task. Results are reported as mean ± std across 32 subjects. **Bold** values indicate the best performance for each model.

Model	Feature Config.	Acc	BA	MF1	WF1	AUC
CustomCNN (SCM)	RGB (PSD+DE+SS)	**0.7401 ± 0.0923**	**0.6144 ± 0.0972**	**0.5987 ± 0.1197**	**0.7065 ± 0.0954**	**0.6811 ± 0.1722**
	DE only	0.6352 ± 0.1040	0.5396 ± 0.0467	0.5227 ± 0.0568	0.6062 ± 0.0857	0.5463 ± 0.0825
	PSD only	0.6669 ± 0.0920	0.5743 ± 0.0532	0.5501 ± 0.0698	0.6310 ± 0.0837	0.5683 ± 0.1075
	SS only	0.6469 ± 0.0955	0.5454 ± 0.0443	0.5209 ± 0.0638	0.6090 ± 0.0833	0.5548 ± 0.0828
	no RGB (raw PSD) ^†^	0.6669 ± 0.0920	0.5743 ± 0.0532	0.5501 ± 0.0698	0.6310 ± 0.0837	0.5683 ± 0.1075
ParallelCNN (BSM)	RGB (PSD+DE+SS)	**0.7428 ± 0.0950**	**0.6478 ± 0.0992**	**0.6447 ± 0.1079**	**0.7259 ± 0.0965**	**0.7049 ± 0.1421**
	DE only	0.6211 ± 0.0901	0.5376 ± 0.0467	0.5320 ± 0.0510	0.6056 ± 0.0832	0.5500 ± 0.0774
	PSD only	0.6374 ± 0.0979	0.5603 ± 0.0664	0.5496 ± 0.0688	0.6194 ± 0.0908	0.5545 ± 0.1064
	SS only	0.6234 ± 0.0888	0.5423 ± 0.0509	0.5346 ± 0.0546	0.6083 ± 0.0783	0.5617 ± 0.0768
	no RGB (raw PSD)	0.6490 ± 0.0978	0.5680 ± 0.0681	0.5550 ± 0.0743	0.6272 ± 0.0932	0.5645 ± 0.1036

^†^ For CustomCNN in its single-branch form, the PSD-only and no-RGB (raw PSD) entries are identical because, in both configurations, the input is a single-channel spatial map without multi-feature concatenation—i.e., SCM-style feature fusion is not applied. See Section 6.1 for details.

**Table 8:** Subject-dependent results (trial-wise 5-fold CV) on DEAP and DREAMER. Mean ± std across subjects. Best per column per dataset per emotion in **bold**.

Dataset	Input	Model	Emotion	Acc	BA	MF1	WF1	AUC
DEAP	SCM	CustomCNN	Arousal	**0.7405 ± 0.0927**	**0.6182 ± 0.1100**	**0.6018 ± 0.1209**	**0.7084 ± 0.0945**	**0.6812 ± 0.1729**
		CustomCNN	Valence	**0.7058 ± 0.0595**	**0.6474 ± 0.0928**	**0.6362 ± 0.1106**	**0.6819 ± 0.0717**	0.6977 ± 0.1199
		EEGNet	Arousal	0.7353 ± 0.0898	0.6069 ± 0.0930	0.5894 ± 0.1166	0.6964 ± 0.0979	0.6557 ± 0.1636
		EEGNet	Valence	0.7021 ± 0.0627	0.6389 ± 0.0876	0.6276 ± 0.1043	0.6769 ± 0.0718	**0.6990 ± 0.1176**
		DeepConvNet	Arousal	0.7088 ± 0.1002	0.5999 ± 0.0916	0.5813 ± 0.1072	0.6789 ± 0.1057	0.6493 ± 0.1424
		DeepConvNet	Valence	0.6832 ± 0.0528	0.6373 ± 0.0949	0.6170 ± 0.1111	0.6606 ± 0.0726	0.6730 ± 0.1284
		ShallowConvNet	Arousal	0.7147 ± 0.0952	0.5980 ± 0.0858	0.5854 ± 0.1048	0.6823 ± 0.1013	0.6469 ± 0.1367
		ShallowConvNet	Valence	0.6778 ± 0.0506	0.6358 ± 0.0649	0.6310 ± 0.0714	0.6642 ± 0.0499	0.6936 ± 0.0787
	BSM	ParallelCNN	Arousal	0.7411 ± 0.0955	0.6345 ± 0.1028	0.6281 ± 0.1176	**0.7181 ± 0.1001**	**0.7077 ± 0.1339**
		ParallelCNN	Valence	**0.7086 ± 0.0473**	**0.6648 ± 0.0672**	**0.6634 ± 0.0693**	**0.6982 ± 0.0461**	**0.7237 ± 0.0798**
		EEGNet	Arousal	**0.7417 ± 0.0968**	0.6174 ± 0.1078	0.5999 ± 0.1303	0.7050 ± 0.1096	0.6481 ± 0.1941
		EEGNet	Valence	0.7011 ± 0.0439	0.6531 ± 0.0759	0.6425 ± 0.0904	0.6839 ± 0.0457	0.6941 ± 0.1120
		DeepConvNet	Arousal	0.7172 ± 0.0935	**0.6450 ± 0.1031**	**0.6369 ± 0.1066**	0.7039 ± 0.0983	0.6841 ± 0.1586
		DeepConvNet	Valence	0.6908 ± 0.0663	0.6596 ± 0.0841	0.6529 ± 0.0904	0.6816 ± 0.0726	0.7166 ± 0.1015
		ShallowConvNet	Arousal	0.7252 ± 0.0983	0.6041 ± 0.0959	0.5923 ± 0.1160	0.6951 ± 0.1043	0.6371 ± 0.1609
		ShallowConvNet	Valence	0.6779 ± 0.0457	0.6339 ± 0.0673	0.6292 ± 0.0709	0.6661 ± 0.0428	0.6883 ± 0.0822
DREAMER	SCM	CustomCNN	Arousal	**0.9109 ± 0.0997**	**0.8869 ± 0.1488**	**0.8771 ± 0.1675**	**0.9015 ± 0.1177**	**0.8036 ± 0.2602**
		CustomCNN	Valence	**0.8631 ± 0.0980**	**0.8278 ± 0.1389**	**0.8197 ± 0.1554**	**0.8489 ± 0.1241**	0.8344 ± 0.1753
		EEGNet	Arousal	0.8947 ± 0.1152	0.8352 ± 0.2003	0.7784 ± 0.2345	0.8707 ± 0.1488	0.7495 ± 0.2341
		EEGNet	Valence	0.8262 ± 0.1015	0.7545 ± 0.1505	0.7277 ± 0.1711	0.8016 ± 0.1305	0.8035 ± 0.1798
		DeepConvNet	Arousal	0.8822 ± 0.1131	0.8302 ± 0.1950	0.7881 ± 0.2230	0.8604 ± 0.1436	0.7556 ± 0.2613
		DeepConvNet	Valence	0.8168 ± 0.1278	0.7432 ± 0.1883	0.7201 ± 0.2172	0.7796 ± 0.1665	0.7402 ± 0.2425
		ShallowConvNet	Arousal	0.9079 ± 0.1075	0.8669 ± 0.1757	0.8588 ± 0.1948	0.8926 ± 0.1332	0.7995 ± 0.2729
		ShallowConvNet	Valence	0.8534 ± 0.1104	0.8210 ± 0.1461	0.7474 ± 0.1814	0.8388 ± 0.1384	**0.8364 ± 0.2088**
	BSM	ParallelCNN	Arousal	**0.9309 ± 0.0922**	**0.9059 ± 0.1478**	**0.8986 ± 0.1663**	**0.9219 ± 0.1128**	**0.9024 ± 0.1245**
		ParallelCNN	Valence	**0.8614 ± 0.1086**	**0.8267 ± 0.1571**	**0.7962 ± 0.1830**	**0.8495 ± 0.1323**	**0.8785 ± 0.1758**
		EEGNet	Arousal	0.8866 ± 0.1215	0.8213 ± 0.2109	0.7789 ± 0.2439	0.8584 ± 0.1570	0.7295 ± 0.2287
		EEGNet	Valence	0.8262 ± 0.1103	0.7688 ± 0.1614	0.7544 ± 0.1867	0.8019 ± 0.1398	0.7807 ± 0.1947
		DeepConvNet	Arousal	0.8996 ± 0.1066	0.8660 ± 0.1698	0.7457 ± 0.2176	0.8859 ± 0.1306	0.8006 ± 0.2244
		DeepConvNet	Valence	0.7899 ± 0.1121	0.7586 ± 0.1368	0.6930 ± 0.1823	0.7783 ± 0.1443	0.7939 ± 0.1807
		ShallowConvNet	Arousal	0.8978 ± 0.1019	0.8835 ± 0.1245	0.8336 ± 0.1699	0.8924 ± 0.1106	0.8848 ± 0.1635
		ShallowConvNet	Valence	0.8266 ± 0.1086	0.7676 ± 0.1687	0.6868 ± 0.1887	0.8009 ± 0.1474	0.7726 ± 0.2366

**Table 9:** Model complexity and inference efficiency. Params and FLOPs in thousands (K); inference time in milliseconds (ms) per sample. GPU memory (Mem) and model storage size measured in MB.

Architecture	Model	Params (K)	GFLOPs	Time (ms)	GPU Mem (MB)	Model Size (MB)
Single-branch (SCM)	CustomCNN	1092.2	0.008764	0.2556	12.53	4.17
	EEGNet	1.6	0.000499	0.2553	12.15	0.01
	DeepConvNet	139.4	0.002136	0.5465	9.07	0.53
	ShallowConvNet	15.8	0.001848	0.1906	9.07	0.06
4-Branch (BSM)	ParallelCNN	2544.7	0.004529	1.5358	17.91	9.71
	4 × EEGNet	2.9	0.000167	1.0601	22.22	0.01
	4 × DeepConvNet	372.8	0.006948	1.7449	9.92	1.42
	4 × ShallowConvNet	62.2	0.001569	0.7128	10.00	0.24

**Table 10:** LOSO cross-validation results on DEAP and DREAMER. Mean ± std across all subjects. Best per column per dataset per emotion in **bold**.

Dataset	Input/Model	Emotion	Acc	BA	MF1	AUC
DEAP	SCM+CustomCNN	Arousal	**0.5778 ± 0.0763**	**0.5163 ± 0.0425**	**0.5003 ± 0.0498**	**0.5230 ± 0.0560**
		Valence	0.5685 ± 0.0368	**0.5369 ± 0.0288**	**0.5263 ± 0.0371**	**0.5463 ± 0.0478**
	SCM+EEGNet	Arousal	0.5586 ± 0.1274	0.4998 ± 0.0321	0.4349 ± 0.0712	0.4924 ± 0.0586
		Valence	0.5607 ± 0.0615	0.5199 ± 0.0357	0.4786 ± 0.0567	0.5363 ± 0.0572
	SCM+DeepConvNet	Arousal	0.5245 ± 0.1019	0.5000 ± 0.0333	0.4504 ± 0.0552	0.5012 ± 0.0619
		Valence	**0.5722 ± 0.0832**	0.5131 ± 0.0315	0.4236 ± 0.0680	0.5236 ± 0.0730
	SCM+ShallowConvNet	Arousal	0.5495 ± 0.0928	0.5011 ± 0.0307	0.4636 ± 0.0425	0.5025 ± 0.0519
		Valence	0.5592 ± 0.0509	0.5229 ± 0.0268	0.4931 ± 0.0494	0.5299 ± 0.0422
	BSM+ParallelCNN	Arousal	**0.5887 ± 0.0825**	**0.5136 ± 0.0337**	**0.4941 ± 0.0435**	**0.5184 ± 0.0532**
		Valence	**0.5740 ± 0.0726**	0.5044 ± 0.0297	0.4837 ± 0.0383	0.5095 ± 0.0468
	BSM+EEGNet	Arousal	0.5795 ± 0.1164	0.5067 ± 0.0339	0.4615 ± 0.0646	0.5093 ± 0.0624
		Valence	0.5686 ± 0.0585	0.5190 ± 0.0358	0.4828 ± 0.0551	0.5260 ± 0.0711
	BSM+DeepConvNet	Arousal	0.5788 ± 0.1101	0.5016 ± 0.0387	0.4598 ± 0.0562	0.5114 ± 0.0633
		Valence	0.5737 ± 0.0586	**0.5372 ± 0.0433**	**0.5121 ± 0.0579**	**0.5525 ± 0.0688**
	BSM+ShallowConvNet	Arousal	0.5697 ± 0.0941	0.5093 ± 0.0299	0.4746 ± 0.0496	0.5094 ± 0.0451
		Valence	0.5681 ± 0.0546	0.5254 ± 0.0319	0.4919 ± 0.0532	0.5372 ± 0.0589
DREAMER	SCM+CustomCNN	Arousal	0.7292 ± 0.1265	0.5469 ± 0.0728	**0.5208 ± 0.1004**	0.5752 ± 0.1353
		Valence	**0.6141 ± 0.0871**	**0.5470 ± 0.0581**	**0.5311 ± 0.0681**	**0.5557 ± 0.0983**
	SCM+EEGNet	Arousal	0.7290 ± 0.1303	**0.5480 ± 0.0778**	0.5007 ± 0.1074	**0.5859 ± 0.1884**
		Valence	0.6001 ± 0.0996	0.5079 ± 0.0581	0.4670 ± 0.0668	0.5081 ± 0.0911
	SCM+DeepConvNet	Arousal	**0.7394 ± 0.1258**	0.5467 ± 0.0843	0.5129 ± 0.1131	0.5784 ± 0.1888
		Valence	0.6000 ± 0.0769	0.5178 ± 0.0395	0.4839 ± 0.0517	0.5224 ± 0.0854
	SCM+ShallowConvNet	Arousal	0.7123 ± 0.1296	0.5207 ± 0.0689	0.4803 ± 0.0983	0.4959 ± 0.1956
		Valence	0.6013 ± 0.0996	0.5154 ± 0.0791	0.4817 ± 0.0933	0.4944 ± 0.1225
	BSM+ParallelCNN	Arousal	**0.7515 ± 0.1284**	**0.5873 ± 0.0923**	**0.5718 ± 0.1242**	**0.6074 ± 0.1758**
		Valence	**0.6555 ± 0.0880**	**0.6005 ± 0.0710**	**0.5870 ± 0.0777**	**0.6183 ± 0.1356**
	BSM+EEGNet	Arousal	0.7246 ± 0.1305	0.5421 ± 0.0651	0.4935 ± 0.0997	0.5297 ± 0.2072
		Valence	0.5932 ± 0.0958	0.5124 ± 0.0566	0.4696 ± 0.0739	0.5062 ± 0.0890
	BSM+DeepConvNet	Arousal	0.7393 ± 0.1196	0.5508 ± 0.0743	0.5203 ± 0.1039	0.5790 ± 0.1616
		Valence	0.6102 ± 0.0803	0.5163 ± 0.0581	0.4877 ± 0.0654	0.5152 ± 0.0940
	BSM+ShallowConvNet	Arousal	0.7171 ± 0.1275	0.5284 ± 0.0741	0.4908 ± 0.1058	0.5163 ± 0.1923
		Valence	0.5927 ± 0.1062	0.5041 ± 0.0560	0.4700 ± 0.0670	0.4913 ± 0.1202

## Data Availability

The DEAP dataset is available from https://www.kaggle.com/datasets/manh123df/deap-dataset/data, and the DREAMER dataset is available from https://zenodo.org/records/546113. Reference links to the original publications: DEAP at https://ieeexplore.ieee.org/document/5871728 and DREAMER at https://ieeexplore.ieee.org/abstract/document/7887697 (all accessed on 1 March 2026).

## Abbreviations

The following abbreviations are used in this manuscript:

EEG	Electroencephalography
CNN	Convolutional neural network
SCM	Spatial concatenation method
BSM	Band-wise stacking method
PSD	Power spectral density
DE	Differential entropy
SS	Spectral strength
RGB	Red, green, and blue
DL	Deep learning
